# Advances in designing Adeno-associated viral vectors for development of anti-HBV gene therapeutics

**DOI:** 10.1186/s12985-021-01715-9

**Published:** 2021-12-13

**Authors:** Njabulo Mnyandu, Shonisani Wendy Limani, Patrick Arbuthnot, Mohube Betty Maepa

**Affiliations:** Wits/SAMRC Antiviral Gene Therapy Research Unit, School of Pathology, Faculty of Health Sciences, University of the Witwatersrand, Johannesburg, South Africa

**Keywords:** AAV, Hepatitis B virus, Capsid engineering, Genome engineering, HBV replication models

## Abstract

Despite the five decades having passed since discovery of the hepatitis B virus (HBV), together with development of an effective anti-HBV vaccine, infection with the virus remains a serious public health problem and results in nearly 900,000 annual deaths worldwide. Current therapies do not eliminate the virus and viral replication typically reactivates after treatment withdrawal. Hence, current endeavours are aimed at developing novel therapies to achieve a functional cure. Nucleic acid-based therapeutic approaches are promising, with several candidates showing excellent potencies in preclinical and early stages of clinical development. However, this class of therapeutics is yet to become part of standard anti-HBV treatment regimens. Obstacles delaying development of gene-based therapies include lack of clinically relevant delivery methods and a paucity of good animal models for preclinical characterisation. Recent studies have demonstrated safety and efficiency of Adeno-associated viral vectors (AAVs) in gene therapy. However, AAVs do have flaws and this has prompted research aimed at improving design of novel and artificially synthesised AAVs. Main goals are to improve liver transduction efficiencies and avoiding immune clearance. Application of AAVs to model HBV replication in vivo is also useful for characterising anti-HBV gene therapeutics. This review summarises recent advances in AAV engineering and their contributions to progress with anti-HBV gene therapy development.

## Background

Hepatitis B virus (HBV) infection is a major public health burden. Approximately 257 million individuals worldwide are chronically infected with the virus and therefore predisposed to cirrhosis, hepatocellular carcinoma (HCC) and liver failure [1]. HBV has a partially double-stranded relaxed circular DNA (rcDNA) genome of approximately 3.2 kilobases (kb) in length. The genome has four overlapping open reading frames (ORFs), namely the polymerase (*P*), core (*C*), surface (*S*) and the *X* ORFs. Expression of HBV genes is controlled by four separate promoters: the basic core, preS1, preS2 and X promoters. The *cis* enhancer elements are enhancer I, located upstream of the X promoter, and enhancer II which is located upstream of the basic core promoter. These regulatory elements are responsible for liver-specific viral gene expression. The *P* ORF encodes a DNA polymerase with priming, reverse transcriptase and RNase H activity. The *C* ORF comprises precore and core regions, collectively termed precore/core. The core region encodes HBV core antigen (HBcAg) which forms the viral capsid, whereas the precore region encodes the HBV e antigen (HBeAg), an immune suppressor and an indicator of active viral replication. The *S* ORF has three initiation codons: preS1, preS2 and S, which respectively initiate translation of large, middle, and small surface proteins. The *X* ORF encodes the regulatory HBx protein, which is essential for viral replication [2].

HBV infection is initiated by low affinity interaction of the infectious Dane particle with glycosaminoglycans located on the hepatocyte surface [3]. Enhanced by the presence of epidermal growth factor, the high affinity binding of myristalyted large surface antigen to the sodium taurocholate co-transporting polypeptide (NTCP) receptor facilitates entry of the nucleocapsid. Mediated by endocytosis, the nucleocapsid is then transferred to the nucleus via the microtubules [4–7]. This is followed by nuclear release of rcDNA, which is then repaired to form covalently closed circular DNA (cccDNA). The cccDNA then serves as the template for transcription of pregenomic RNA (pgRNA) and viral protein-encoding mRNAs (reviewed in [8]). HBx binds the DDB domain of ubquitin ligase 1 to render structural maintenance of chromosomes protein 5**/**6 unstable and thereby facilitates HBV gene expression [9–11]. Translation of the precore/core RNA produces HBcAg. Encapsidation of the pgRNA is followed by its reverse transcription. The mature nucleocapsid is then transported to the nucleus to maintain cccDNA pools or acquires a surface antigen-containing envelope to form intact virions (Dane particles) following secretion via the endoplasmic reticulum.

Current treatment of HBV infection requires long-term therapy and reduces severe complications and death, but rarely eliminates the virus. This is as a result of inability to clear the stable cccDNA episome from infected hepatocytes and antigenemia-mediated exhaustion of HBV-specific CD8^+^ T cells and B cells [12, 13]. Hence developing a cure for HBV infection is a priority. Recently, gene-based and combinatorial strategies targeting multiple steps in the HBV replication cycle have shown promise. The potential of gene-based strategies for eliminating cccDNA and reducing antigenemia has been demonstrated in preclinical studies (reviewed in [14]). These gene-based approaches include gene editing and gene silencing. Strategies have applied technology based on clustered regulatory interspaced short palindromic repeats (CRISPR)/CRISPR associated (Cas) systems, transcription activator-like effector nucleases (TALENS) and RNA interference (RNAi) to inhibit HBV gene expression (reviewed in [15]). The current challenges of anti-HBV gene therapeutics include difficulties with obtaining a clinically relevant vector for hepatic transgene delivery and the paucity of suitable animal models that simulate natural HBV infection.

Recent US Food and Drug Administration (FDA) approval of AAV-based therapies Zolgesma and Luxturna reinforces a well-established biosafety profile and efficacy of AAVs for human application [16–18]. AAV mediation of hepatic transgene expression is also now well established [19–21]. Moreover, HBV infection enhances AAV transduction of hepatocytes [22]. Despite these appealing features, use of AAVs has not been without its challenges. These include low packaging capacity, reduced transduction efficiencies in specific tissues, induction of CD8^+^ T cell responses and clearance by pre-existing immunity. This review focuses on recent progress with modifying AAVs and their contribution to advancing anti-HBV gene therapy.

## Biology of Adeno-associated viruses

AAVs are small viruses that cannot replicate on their own, but depend on co-infection with other viruses such as adenoviruses or herpes simplex virus or vaccina virus or human papilloma virus (reviewed in [23]). Twelve AAV serotypes have been identified to date. The non-enveloped viral capsid, comprising VP1, VP2 and VP3, has conserved eight-stranded β-barrel motifs, an α-helix and nine variable regions that confer AAV tropism diversity [24]. Multiple viral surface sites have been mapped and characterised as T-cell epitopes, immunogenic motifs and monoclonal antibody docking sites [25–27].

The AAV capsid encases the genome that comprises linear single-stranded DNA (ssDNA) of about 4.7 kb. The genome consists of *Cap* and *Rep* open reading frames (ORFs) flanked by 145 bp T-shaped hairpin inverted terminal repeats (ITRs). The ITRs are made up of three complementary palindromes (A–A′, B–B′ & C–C′), a single non palindromic region (D) and *cis*-acting elements, which are the Rep protein binding element (RBE) and terminal resolution site (trs). Upon host cell entry, AAVs enter latency. In the presence of a helper virus AAVs express their genes from a trio of promoters (p40, p5 and p15) and becomes lytic (reviewed in [23]). Transcription from all the three promoters is terminated by a common poly-adenylation signal. Expression from the p40 promoter produces the three capsid proteins (VP1, VP2 and VP3) and an assembly activating protein (AAP). The capsid proteins assemble in 1:1:10 ratio of VP1:VP2:VP3 to form an icosahedral capsid while AAP mediates capsid assembly. *Rep* gene expression is driven from p5 & p19 promoters to produce two large (Rep78 and Rep68) and two small (Rep52 and Rep40) Rep proteins (reviewed in [28, 29]).

AAV2 infection is initiated by binding to the heparin sulphate proteoglycan receptor and a co-receptor fibroblast growth factor receptor. Endocytosis through clathrin-coated vesicles then mediates viral entry [30, 31]. In the absence of a helper virus, AAV gene expression is limited and the genome mainly persists episomally. Less frequently, while various integration sites exist for AAV such as AAVS2 or AAVS3, AAV DNA integrates preferentially into the AAVS1 site of the host genome [32, 33]. In the presence of a helper virus, expression from the *Rep* ORF is activated and enables AAV genome rescue. DNA polymerase mediates second strand synthesis using one ITR as a primer to produce a double stranded DNA (dsDNA). Together with Rep78/68 and several host factors, DNA polymerase uses the dsDNA as a template for re-initiation and polymerisation from one end to generate a double stranded full-length genome and displace a single stranded full-length genome. The double stranded genome serves as a template for further rounds of replication, while the rep proteins mediate ssDNA loading into the capsid. An active release pathway of AAV particles following viral assembly remains to be described (reviewed in [34, 35]).

### Engineering AAV genome for delivery of anti-HBV sequences

**Fig. 1:**
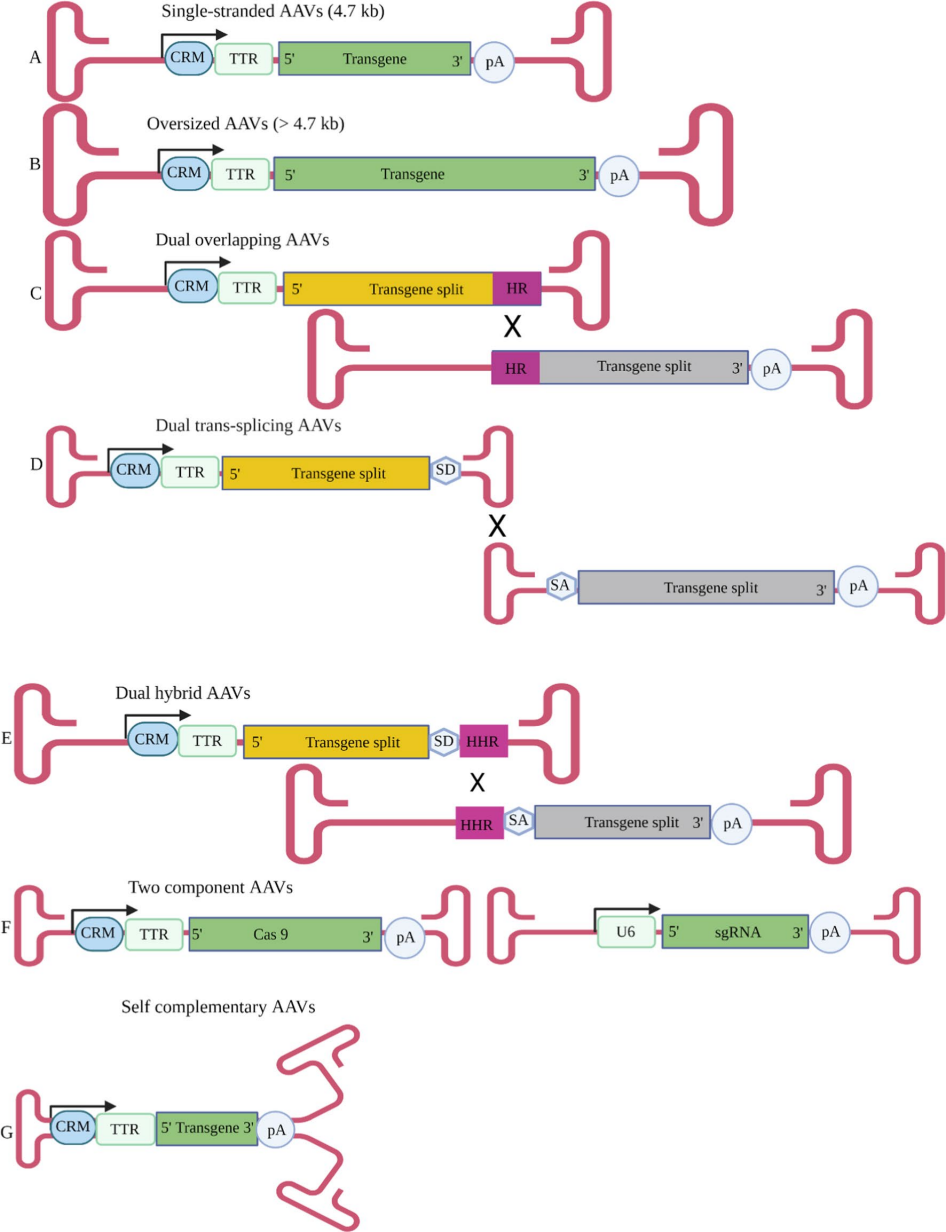
AAV genome modifications. **A** A native single stranded AAV (ssAAV) genome, about 4.7 kb in size, customised for hepatic-specific transgene expression with use of liver-specific promoters such as mouse Transthyretin (TTR) or human TTR promoters. The *cis*-regulatory modules (CRM) to enhance expression and polyadenylation signal (pA) are also indicated. **B** Oversized AAV vector genome (bigger than 4.7.kb). **C** Dual overlapping AAV genomes with 5′ and 3′ transgene splits are indicated by yellow or grey boxes respectively. Pink boxes and a cross indicate homologous regions (HR) and a homologous recombination site respectively. **D** Dual trans-splicing AAV genomes. Splicing donor (SD) and splicing acceptor (SA) sequence indicated downstream or upstream of the transgene splits. The cross indicate the site for concatemerisation. **E** Dual hybrid AAV genomes. Highly homologous recombineering (HHR) region is indicated by pink boxes. **F** Two component vectors, each expressing Cas 9 or sgRNA. **G** A self-complementary AAV (scAAV) genome with half the size (about 2.35 kbp)

For AAV vector production, the ITR sequences are retained but the promoter sequences, *rep* and *cap* genes are removed to accommodate transgene cassettes (Fig. 1A). This generally allows insertion of a maximum of about 5 kb sequences into the vectors. Although this is enough for smaller anti-HBV effectors, such as RNAi activators, it precludes delivery of larger anti-HBV CRISPR/Cas and TALEN sequences. Early studies described successful production of oversized AAV vectors and packaging of genomes larger than 5 kb (Fig. 1B) [36–40]. However, during viral production AAVs with heterogeneous genome sizes with truncations are often produced from these oversized genomes. Upon transduction, large AAV genomes may be reconstituted by concatemerisation, but reconstitution using the truncated genomes is highly inefficient and not desirable for clinical application.

Based on ability of AAV genomes to concatemerise and serve as substrates for homologous recombination, another strategy to increase tansgene capacity entails use of dual AAV vectors [40, 41]. The most commonly used are dual overlapping vectors, dual trans-splicing vectors and dual hybrid vectors (Fig. 1C–E). The design is to split the expression cassette into two parts, each contained in an AAV, and the intact transgene is reconstituted in a cell after transduction by homologous recombination or concatamerisation. As with oversized vectors, reconstitution of dual vectors is inefficient. This results in poor transduction efficacies and a requirement for high vector doses to achieve therapeutically relevant effects [37, 42–44].

Recent studies have taken advantage of CRISPR/Cas systems being made up of two components, i.e. the nuclease and the single guide RNA (sgRNA) (Fig. 1F). These may be expressed on separate AAVs and in combination effect DNA cleavage upon transduction of a cell by the two vectors [45]. With recent availability of smaller nucleases, a single AAV can now be used to deliver both the nuclease- and sgRNA-encoding sequences to effect cccDNA cleavage [46–49]. TALEN activity requires two subunits, each encoded by DNA of at least 3 kb, to effect dsDNA cleavage. Although the evidence is scant, two component vector systems should be applicable to delivering sequences that together constitute complete anti-HBV TALENS.

A requirement to convert an ssDNA AAV genome to a dsDNA before transgene expression is a limiting step of AAV-based gene transfer. For quicker transgene expression, the trs site may be mutated to inhibit terminal resolution. This results in AAVs bearing a long hairpin loop molecule with complementary duplex strands referred to as self-complementary AAVs (scAAVs, Fig. 1G). Although this reduces packaging capacity by half, scAAVs bypass the requirement for second strand synthesis with consequent faster and higher transgene expression [35, 50].

AAVs are known to infect a diverse range of tissues, which might lead to undesirable off-target transgene expression [51]. Hepatic tissue-specific expression of anti-HBV gene therapies can be achieved by placing transgene expression under control of liver-specific promoters, such as Transthyterin (TTR) or mouse Transthyretin (mTTR) (Fig. 1) [19, 20]. Liver-specific regulatory elements derived from core domains of human apolipoprotein hepatic control region, human α-1-antitrypsin and hybrid liver promoters successfully drive factor IX expression in the liver [52, 53] and may be applicable to anti-HBV gene therapy. When in silico identified evolutionary conserved hepatocyte-specific *cis-*regulatory modules (CRMs) were incorporated into scAAVs, up to 100-fold higher transgene expression was achieved when compared to scAAVs cassettes containing the TTR promoter (Fig. 1) [54].

### AAV capsid engineering for improved transduction and evasion of pre-existing immunity

The requirement for a high AAV dose to achieve therapeutic effects in non-human primates has been reported to result in death [55]. Hence, production of AAV capsids that achieve high transduction efficiencies at low dose is an important goal of the field. AAV capsid structural properties determine vector tropism, immune detection and transduction efficiency. Hence, manipulation of capsid architecture is central to enhancing the vectors’ therapeutic efficacy. High prevalence of pre-existing AAV-specific antibodies in humans, which limits AAV-mediated gene transfer, is another major reason for investigating utility of AAV capsid modification [56, 57]. In addition, proteasomal degradation, breakdown of capsids following endosomal escape and MHC1 presentation of AAV peptides with cell-mediated elimination of infected hepatocytes result in poor transgene expression [58–61]. Approaches have mainly involved rational design or directed evolution to modify AAV capsids. The former relies on prior knowledge of capsid architecture and intracellular trafficking of AAVs. By contrast, directed evolution utilises stringent selection methods to concentrate and confer advantageous and beneficial traits on a vector.

#### Rational designs of capsids

**Fig. 2:**
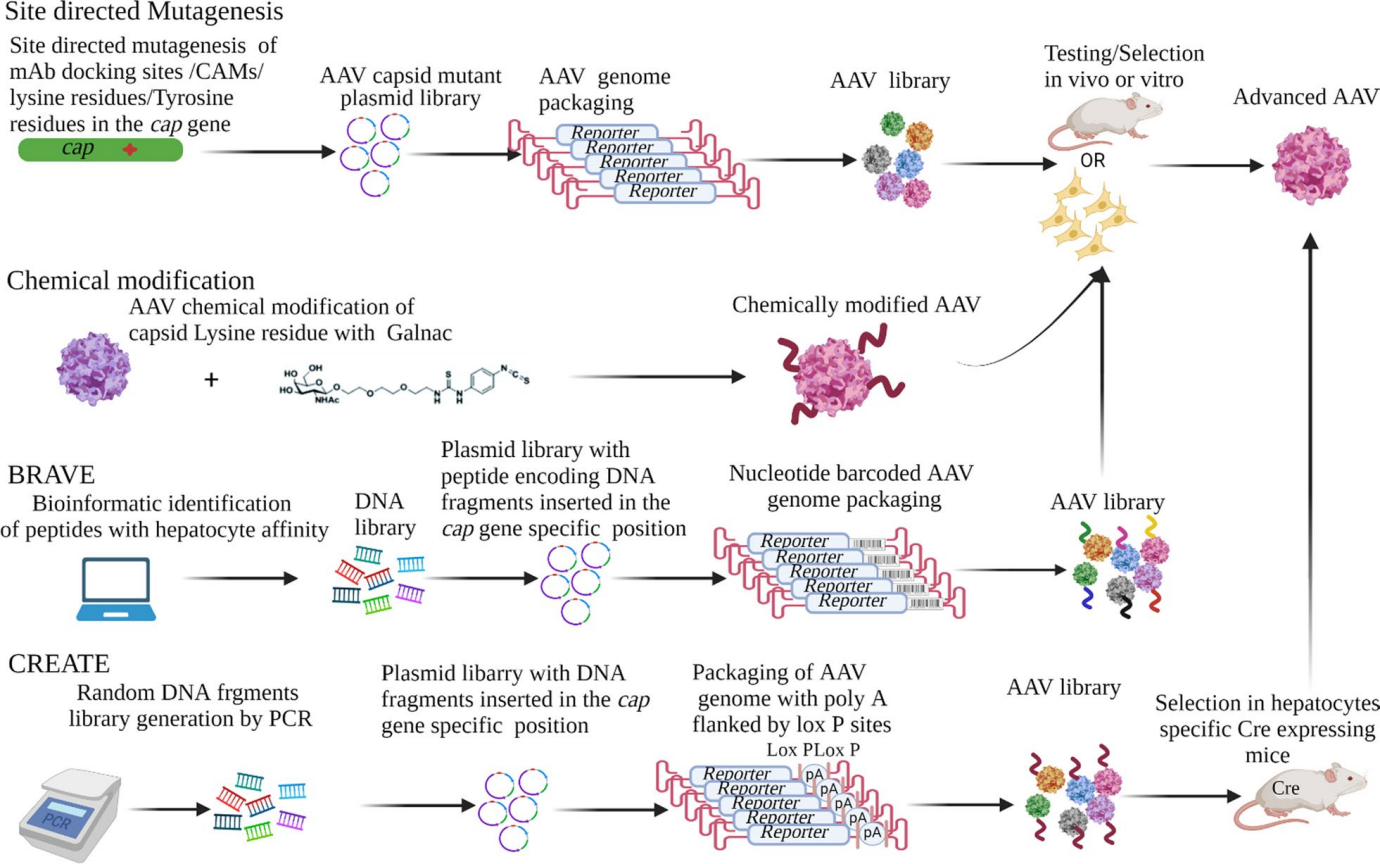
Rational strategies of AAV capsid modification. Monoclonal antibody docking sites, capsid antigenic motifs (CAM) and lysine residues associated with ubiquitination, neddylation, SUMOylation or glycosylation in the capsid are mutated by site directed mutagenesis. Mutated capsid sequences are cloned in to a Rep-encoding plasmid to create a plasmid library that is then used to package a reporter encoding AAV genome. The AAV library is then put through several rounds of selection in vivo or in cell culture to enrich for AAV variants with desirable traits such as hepatocyte transduction and immune evasion. Chemical modification with compounds with hepatocyte affinity e.g. GalNAc, are attached to the AAV capsid to generate AAVs with enhanced hepatocyte transduction. Barcoded rational AAV vector evolution (BRAVE) involves bioinformatics identification of proteins with hepatocyte affinity. A DNA library encoding peptide fragments is generated and inserted in the specific positions of the capsid sequence within the Rep-encoding plasmid to produce a mutant capsid plasmid library. This is followed by packaging of the reporter encoding AAV genome bearing a unique nucleotide barcode sequence. Cre-recombination-based AAV targeted evolution (CREATE) involves insertion of random PCR generated fragments between specific capsid gene positions within the Rep-encoding plasmid to produce mutant capsid plasmid library. A reporter-encoding AAV genome with the poly A signal flanked by lox P sites is then packaged. The AAV library is selected in hepatocytes specific Cre expressing mice

Several AAV variants with desirable features have been developed by using different rational design strategies. Some of the AAV strategies discussed below were used to develop variants for transduction of non-liver-derived cells. However, these approaches can be applied to improve efficiency of liver-targeting vectors. Docking sites of monoclonal antibodies (mAbs) or capsid antigenic motifs (CAMs) located in capsid variable regions (VR) serve as targets of capsid modification to avoid neutralising antibody (NAb) recognition. When these CAMs were mutated to produce libraries of novel AAV capsid variants (AAV-CAMs) followed by iterative rounds of selection in endothelial cells, antigenically advanced capsids were identified [62, 63] (Fig. 2).

Using a method called barcoded rational AAV vector evolution (BRAVE), rational design and screening for AAV variants that ably transduce cells of the central nervous system (CNS) were identified. To build an AAV library, proteins with synaptic affinity were identified using bioinformatics, and their peptide fragments inserted at a specific position to produce mutant capsids. AAV genomes bearing unique nucleotide barcode sequences were packaged into the mutant capsids to enable identification of individual capsid structures [64, 65]. Another method designated Cre-recombination-based AAV targeted evolution (CREATE) was used to generate vectors capable of transducing the CNS. The 7-mer random PCR-generated fragments were inserted between sequences encoding amino acid residues 588 and 589 of the capsid gene. The downstream poly A signal was flanked by lox P sites. AAV library administration in a cell type specific cre transgenic mice allowed inversion of the poly A signal, creating a sequence that could be amplified using pre-designed PCR primers. This led to the isolation of capsids that were capable of infecting cre-expressing cells (Fig. 2) [66].

Phosphorylation of AAV tyrosine or lysine residues by host cellular machinery leads to AAV capsid degradation by the ubiquitin–proteasome pathway [61, 67]. Other post-translational modifications such as glycosylation, SUMOylation, and neddylation also impact on viral transduction. Glycosylation facilitates viral cell entry, trafficking to the nucleus, virulence and immune evasion (reviewed in [68]). As with ubiquitination, neddylation and SUMOylation form reversible covalent attachments at lysine residues. These modifications affect protein stability, subcellular localisation, structure and function to inhibit AAV transduction of cells [69, 70]. Mutation or chemical modification of lysine residues in AAV2 or AAV8 capsids where glycosylation, neddylation or SUMOylation occurs resulted in higher transgene expression and decreased interaction of the AAV with NAbs (Fig. 2) [71–74].

#### Directed evolution designs of novel capsids

**Fig. 3:**
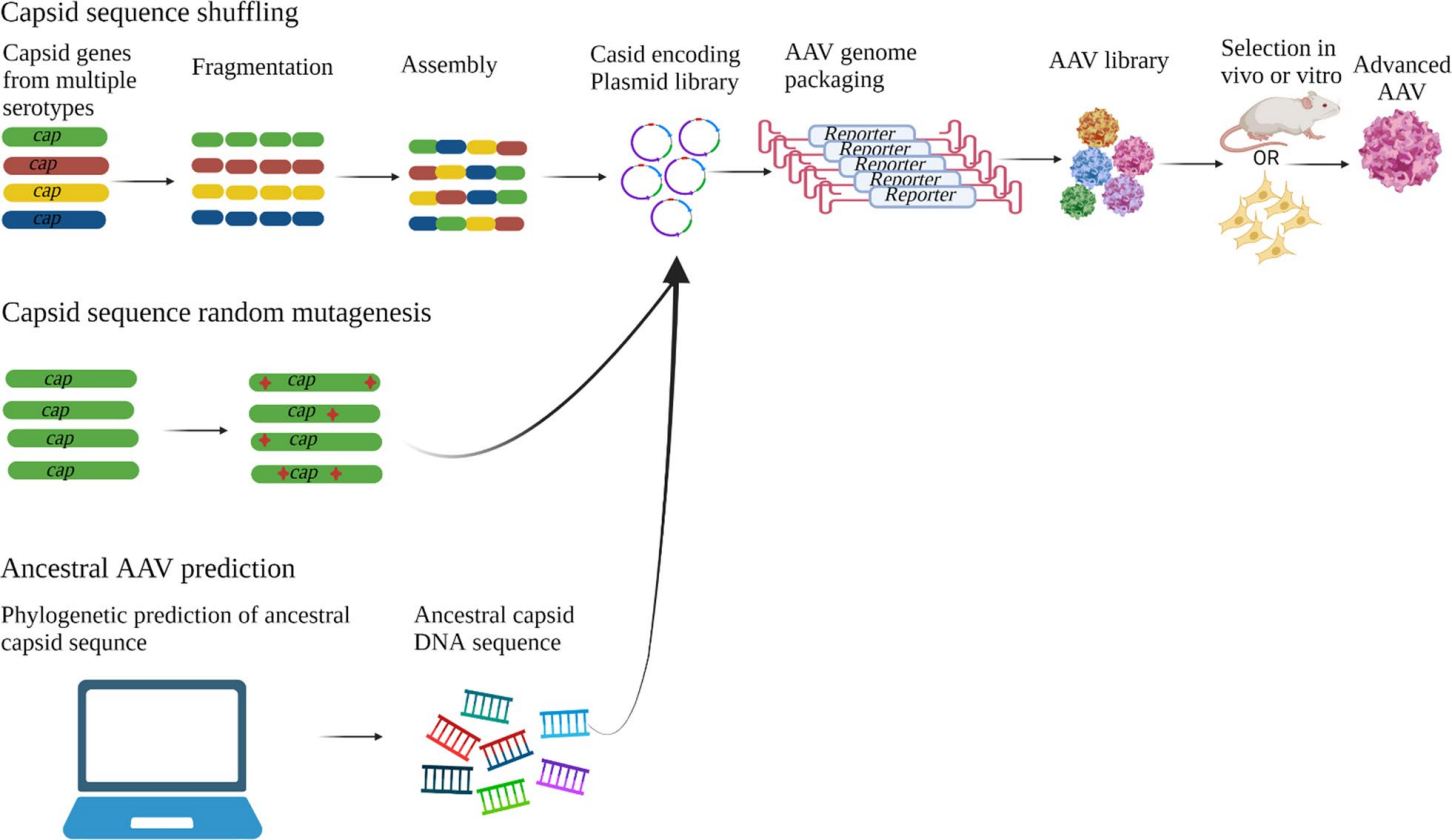
Directed evolution strategies of AAV capsid modification. DNA shuffling of various serotypes’ *cap* sequences is achieved by fragmenting the capsid sequences and assembly to create hybrid capsid sequences, which can be cloned in the a Rep-encoding plasmid to create an AAV capsid plasmid library. The plasmid is then used to package a reporter-expressing AAV genome followed by selection in vitro or in vivo. Capsid sequence random mutagenesis can be performed to create a mutant capsid library, which is cloned in to a Rep-encoding plasmid to create an AAV library that can be selected. Ancestral capsid sequences, predicted using bioinformatic tools, can be synthesized in vitro and used to produce an ancestral capsid plasmid library and an AAV library before selection

DNA shuffling of capsid-encoding sequences from multiple AAV serotypes has also been used to generate libraries. This approach has been used to identify capsids with improved hepatocyte transduction capabilities (Fig. 3) [75–77]. Libraries of AAVs may also be generated by random capsid sequence mutagenesis. Stringent selection of these mutant libraries in vitro and in chimeric murine livers identified variants with improved transduction efficiencies (Fig. 3) [78, 79]. Another strategy employed phylogenetic techniques to predict ancestral AAV capsid sequences that mediated higher transgene expression than natural AAV serotypes [80] (Fig. 3). Studies carried out in vivo on small and large animals identified another antigenically distinct and antibody-evading ancestral AAV vector that efficiently transduced a variety of cells including the liver [81–86].

### How has anti-HBV gene therapy designs benefited from the advances in AAV vector developments?

#### Application of improved AAVs to deliver anti-HBV gene therapeutics

The higher liver transgene expression from AAV8-pseudotyped scAAVs enables use of low vector doses. Also, the dsDNA nature of the scAAV genome makes it more stable [52, 87–89]. These properties are favourable for targeting chronic viral infections such as are caused by HBV. The feasibility of delivering anti-HBV RNAi activators using scAAVs is well documented [90, 91]. In mice, following a single dose of AAV8-pseudotyped scAAVs a reduction of HBV replication markers was observed over 10 months [19]. Targeting both HBV and the host factors that mediate fibrosis with scAAVs improves therapeutic efficacy [92]. Supporting this combinatorial approach is the observation that scAAVs used to co-deliver an RNAi effector against HBV and Argonaut2, the rate limiting host factor in the RNAi pathway or a RNAi activator sense strand targeted decoy, improves safety, specificity, and efficacy [20, 93, 94]*.*

Because of the limiting packaging capacity of scAAVs, several studies have used ssAAVs for delivery of a smaller *Staphylococcus aureus* Cas9 (SaCas9) with single or combination of guides targeting several coordinates in the cccDNA. These studies showed significant decline in markers of HBV replication in cultures and in nice [46–48]. A recent study has illustrated that using ssAAVs engineered to express saCas9 from a chimeric liver-specific promoter resulted in preferential liver expression and superior suppression of HBV replication in mice [95]. One study that used scAAVs to deliver anti-HBV gene editors co-transduced cells with two scAAVs, each carrying one ZFN monomer against HBV *Pol*, *C* or *X* genes. scAAVs against *Pol* resulted in near undetectable levels of HBV replication makers [96].

The anti-HBV gene therapy field has not yet fully capitalised on the availability of modified or synthetic AAV capsids described above. However, these developments, especially the genetically modified and synthetic AAV capsids that shows high liver transduction efficiencies, are alluring and promise to bring positive outcomes to HBV treatment.

#### Application of AAVs to model chronic HBV infection

The plethora of AAV serotypes, either extant or artificially synthesized and recent discovery of various receptors and co-receptors that facilitate AAV binding, are valuable tools to model HBV replication [97, 98]. These models are key to evaluating novel anti-HBV therapeutic interventions before clinical translation. Despite impressive progress with understanding the molecular biology of HBV, an easily accessible model that can recapitulate all stages of HBV remains elusive. Although chimpanzees are susceptible to HBV infection, and their immune responses are similar to those observed in humans, high cost and ethical concerns limit use of these animals in research (reviewed in [99]). Models using species-specific hepatitis strains such as duck hepatitis B virus and woodchuck hepatitis virus are limited by infection mechanisms and disease manifestations in these models that differ from natural HBV infection (Table 1) [100, 101].

**Table 1:** Animal models of HBV chronic infection

Cell culture/animal model	Immune response	HBV antigen expression	Infection/replication	cccDNA formation	Fibrosis	Liver injury	References
Chimpanzee	Immuno-competent	Yes	Infection	Yes	Yes	Yes	[99]
Woodchuck	Immuno-competent	Yes	Infection	Yes	Yes	Yes	[100, 101]
Duck HBV	Immuno-competent	Yes	Infection	Yes	Yes	Yes	[101]
Human liver Chimeric mouse model	Immuno-deficient	Yes	Infection	Yes	Yes	Yes	[103–105]
HBV Transgenic mouse model	Immuno-competent	Yes	Replication	No	No	No	[19, 122]
AAV-HBV mouse model	Immuno- competent	Yes	Replication	No	Yes	Yes	[113–115]
hNTCP expressing Rhesus macaques	Immuno-competent	Yes	Infection	Yes	–	Yes	[121, 123]

Key: – not known

Mouse models to simulate HBV replication remain the most accessible and commonly used. Chimeric mice with livers engrafted with human hepatocytes are valuable, but use of these animals is limited by their extreme immuno-deficiency and difficulties with maintaining hepatocyte function over long periods of time [102–105]. Transgenic mice with integrated DNA comprising greater-than-genome-length HBV sequences mimic chronic HBV replication [106–108]. However, transgenic mice show inter-individual variability, variation of HBV replication markers over time and high markers of HBV replication that often exceed those of natural HBV infection (Table 1) [19, 109].

Use of recombinant adenoviruses and AAVs to deliver greater-than-genome-length HBV sequences has also been explored to simulate HBV replication in vivo. Adenoviral vectors efficiently induce HBV replication in mice [110–112]. However, transduction results in a strong immune response to the adenoviral vectors and early clearance of transduced hepatocytes. Three studies using an AAV8-carrying replication-competent greater-than-genome-length HBV genotype D (AAV-HBV model) demonstrated the potential of these vectors for simulation of chronic HBV infection [113–115]. HBsAg, HBeAg and HBcAg expression, accompanied by hepatitis B virion production over a period of up to 16 months, were observed. Production of anti-HBcAg antibodies, but not anti-HBsAg and HBeAg antibodies which is a phenomenon observed in HBV chronic carriers, was also demonstrated. Interestingly, between 12 and 16 months, mice developed features of HCC and elevated makers of liver injury. Liver fibrosis, chronic liver injury and minimal or no acute inflammatory responses were observed in these mice. Given that T cell exhaustion is a well-documented feature of chronic HBV infection, the lack of a significant immune response in these mice is perhaps not surprising (Table 1) [116, 117]. Although the mechanism of HCC, fibrosis and chronic liver damage in AAV-HBV murine models remains to be established, expression of HBV antigens such as HBx and HBsAg, together with AAV-mediated HBV DNA integration may be the contributing factors. Interestingly, recent studies showed that the AAV-HBV model based on either HBV serotype A, B, C or D may result in formation of cccDNA in murine hepatocytes [118, 119]. Although the replication intermediate was lost over time in transduced livers, the sequence and functionality was not distinguishable from cccDNA derived from natural HBV infection. The mechanism of its formation is not clear, however the cccDNA is HBV replication-independent and originates from intramolecular recombination of the HBV genome ends [119].

Another model recently established in non-human primates used AAV8 vectors expressing human NTCP (AAV8-hNTCP) [120, 121]. The study also employed helper-dependent adenoviral vectors (HDAds) to deliver sequences encoding hNTCP. Rhesus macaques, naturally not infectable by HBV, were injected with hNTCP-expressing vectors and then infected with HBV. HBV gene expression and HBV replication intermediates were detected over a period of 42 weeks. Moreover, markers of liver injury and T cell responses to HBV antigens were reported. Importantly the essential replication intermediate comprising cccDNA could also be detected in these macaques. Although a higher AAV vector dose was required and HDAd vectors promoted superior hNTCP expression and HBV infection, adenoviral vectors are limited by their high immune stimulation and the resultant short-term transgene expression. Hence, administration of immunosuppressants before injecting HDAds was required to prolong HBV gene expression. This makes HDAd-based models less favourable for mimicking chronic HBV infection.

## Conclusion

Studies described here show that progress with the design of improved AAV vectors will assist with addressing challenges facing development of anti-HBV gene therapy. Improved liver transduction will make it possible to administer lower vector doses to achieve clinically relevant therapeutic outcomes. Engineering AAVs to produce vectors that can evade systemic and cellular trafficking hurdles to deliver anti-HBV payloads to target cells expands the toolbox of gene therapy for the viral infection. Harnessing AAVs’ liver tropism to model HBV replication also shows potential. Although murine models remain the most accessible and simple, demonstration that AAVs may be used to make non-human primates susceptible to HBV infection is significant. Collectively these developments will facilitate clinical translation of AAV-based, as well as other potentially curative therapies, to eliminate chronic HBV infection.

## Data Availability

Not applicable.

## Abbreviations

AAV: Adeno-associated virus
BCP: Basic core promoter
bp: Base pairs
C: Core
Cas: CRISPR associated
cccDNA: Covalently closed circular DNA
CRISPR: Clustered regulatory interspaced short palindromic repeats
HBcAg: Hepatitis B core antigen
HBeAg: Hepatitis B E antigen
HBsAg: Hepatitis B surface antigen
HBV: Hepatitis B virus
HBx: Hepatitis B X protein
HCC: Hepatocellular carcinoma
ITR: Inverted terminal repeats
Kb: Kilobases
NTCP: Sodium taurocholate polypeptide
Orfs: Open reading frame
P: Polymerase
RBE: Rep binding element
rcDNA: Relaxed circular DNA
RNAi: RNA interference
S: Surface
TALENS: Transcription activator-like effector nucleases
Trs: Terminal resolution site
VR: Capsid variable regions

## References

[1] WHO (2017). Global hepatitis report, 2017.

[2] Lucifora J, Arzberger S, Durantel D, Belloni L, Strubin M, Levrero M (2011). Hepatitis B virus X protein is essential to initiate and maintain virus replication after infection. J Hepatol.

[3] Sureau C, Salisse J (2013). A conformational heparan sulfate binding site essential to infectivity overlaps with the conserved hepatitis B virus a-determinant. Hepatology.

[4] Yan H, Zhong G, Xu G, He W, Jing Z, Gao Z (2012). Sodium taurocholate cotransporting polypeptide is a functional receptor for human hepatitis B and D virus. Elife.

[5] Ni Y, Lempp FA, Mehrle S, Nkongolo S, Kaufman C, Falth M (2014). Hepatitis B and D viruses exploit sodium taurocholate co-transporting polypeptide for species-specific entry into hepatocytes. Gastroenterology.

[6] Iwamoto M, Saso W, Sugiyama R, Ishii K, Ohki M, Nagamori S (2019). Epidermal growth factor receptor is a host-entry cofactor triggering hepatitis B virus internalization. Proc Natl Acad Sci U S A.

[7] Iwamoto M, Saso W, Nishioka K, Ohashi H, Sugiyama R, Ryo A (2020). The machinery for endocytosis of epidermal growth factor receptor coordinates the transport of incoming hepatitis B virus to the endosomal network. J Biol Chem.

[8] Nassal M (2015). HBV cccDNA: viral persistence reservoir and key obstacle for a cure of chronic hepatitis B. Gut.

[9] Decorsiere A, Mueller H, van Breugel PC, Abdul F, Gerossier L, Beran RK (2016). Hepatitis B virus X protein identifies the Smc5/6 complex as a host restriction factor. Nature.

[10] Murphy CM, Xu Y, Li F, Nio K, Reszka-Blanco N, Li X (2016). Hepatitis B virus X protein promotes degradation of SMC5/6 to enhance HBV replication. Cell Rep.

[11] Allweiss L, Giersch K, Pirosu A, Volz T, Muench RC, Beran RK (2021). Therapeutic shutdown of HBV transcripts promotes reappearance of the SMC5/6 complex and silencing of the viral genome in vivo. Gut.

[12] Fisicaro P, Barili V, Montanini B, Acerbi G, Ferracin M, Guerrieri F (2017). Targeting mitochondrial dysfunction can restore antiviral activity of exhausted HBV-specific CD8 T cells in chronic hepatitis B. Nat Med.

[13] Kurktschiev PD, Raziorrouh B, Schraut W, Backmund M, Wachtler M, Wendtner CM (2014). Dysfunctional CD8+ T cells in hepatitis B and C are characterized by a lack of antigen-specific T-bet induction. J Exp Med.

[14] Bloom K, Maepa MB, Ely A, Arbuthnot P (2018). Gene therapy for chronic HBV—can we eliminate cccDNA?. Genes.

[15] Maepa MB, Jacobs R, van den Berg F, Arbuthnot P (2020). Recent developments with advancing gene therapy to treat chronic infection with hepatitis B virus. Curr Opin HIV AIDS.

[16] Kassner U, Hollstein T, Grenkowitz T, Wuhle-Demuth M, Salewsky B, Demuth I (2018). Gene therapy in lipoprotein lipase deficiency: case report on the first patient treated with alipogene tiparvovec under daily practice conditions. Hum Gene Ther.

[17] Kirschner J, Butoianu N, Goemans N, Haberlova J, Kostera-Pruszczyk A, Mercuri E (2020). European ad hoc consensus statement on gene replacement therapy for spinal muscular atrophy. Eur J Paediatr Neurol.

[18] Prado DA, Acosta-Acero M, Maldonado RS (2020). Gene therapy beyond luxturna: a new horizon of the treatment for inherited retinal disease. Curr Opin Ophthalmol.

[19] Maepa MB, Ely A, Grayson W, Arbuthnot P (2017). Sustained inhibition of HBV replication in vivo after systemic injection of AAVs encoding artificial antiviral primary MicroRNAs. Mol Ther Nucleic Acids.

[20] Michler T, Grosse S, Mockenhaupt S, Roder N, Stuckler F, Knapp B (2016). Blocking sense-strand activity improves potency, safety and specificity of anti-hepatitis B virus short hairpin RNA. EMBO Mol Med.

[21] Ye L, Kan F, Yan T, Cao J, Zhang L, Wu Z (2017). Enhanced antiviral and antifibrotic effects of short hairpin RNAs targeting HBV and TGF-beta in HBV-persistent mice. Sci Rep.

[22] Hosel M, Lucifora J, Michler T, Holz G, Gruffaz M, Stahnke S (2014). Hepatitis B virus infection enhances susceptibility toward adeno-associated viral vector transduction in vitro and in vivo. Hepatology.

[23] Geoffroy M-C, Salvetti A (2005). Helper functions required for wild type and recombinant adeno-associated virus growth. Curr Gene Ther.

[24] Xie Q, Bu W, Bhatia S, Hare J, Somasundaram T, Azzi A (2002). The atomic structure of adeno-associated virus (AAV-2), a vector for human gene therapy. Proc Natl Acad Sci U S A.

[25] DiMattia MA, Nam HJ, Van Vliet K, Mitchell M, Bennett A, Gurda BL (2012). Structural insight into the unique properties of adeno-associated virus serotype 9. J Virol.

[26] Mingozzi F, Maus MV, Hui DJ, Sabatino DE, Murphy SL, Rasko JE (2007). CD8(+) T-cell responses to adeno-associated virus capsid in humans. Nat Med.

[27] Vandenberghe LH, Wang L, Somanathan S, Zhi Y, Figueredo J, Calcedo R (2006). Heparin binding directs activation of T cells against adeno-associated virus serotype 2 capsid. Nat Med.

[28] Kotterman MA, Schaffer DV (2014). Engineering adeno-associated viruses for clinical gene therapy. Nat Rev Genet.

[29] Li C, Samulski RJ (2020). Engineering adeno-associated virus vectors for gene therapy. Nat Rev Genet.

[30] Joo K, Fang Y, Liu Y, Xiao L, Gu Z, Tai A, Lee C (2011). Enhanced real-time monitoring of adeno-associated virus trafficking by virus-quantum dot conjugates. ACS Nano.

[31] Liu Y, Joo KI, Wang P (2013). Endocytic processing of adeno-associated virus type 8 vectors for transduction of target cells. Gene Ther.

[32] Hüser D, Gogol-Döring A, Lutter T, Weger S, Winter K, Hammer E-M (2010). Integration preferences of wildtype AAV-2 for consensus Rep-binding sites at numerous loci in the human genome. PLOS Pathog.

[33] Kotin RM, Siniscalco M, Samulski RJ, Zhu XD, Hunter L, Laughlin CA (1990). Site-specific integration by adeno-associated virus. Proc Natl Acad Sci U S A.

[34] Weitzman MD, Linden RM (2011). Adeno-associated virus biology. Methods Mol Biol.

[35] McCarty DM (2008). Self-complementary AAV vectors; advances and applications. Mol Ther.

[36] Nambiar B, Cornell Sookdeo C, Berthelette P, Jackson R, Piraino S, Burnham B (2017). Characteristics of minimally oversized adeno-associated virus vectors encoding human factor VIII generated using producer cell lines and triple transfection. Hum Gene Ther Methods.

[37] McClements ME, Charbel Issa P, Blouin V, MacLaren RE (2016). A fragmented adeno-associated viral dual vector strategy for treatment of diseases caused by mutations in large genes leads to expression of hybrid transcripts. J Genet Syndr Gene Ther.

[38] Hirsch ML, Wolf SJ, Samulski RJ (2016). Delivering transgenic DNA exceeding the carrying capacity of AAV vectors. Methods Mol Biol.

[39] Dyka FM, Boye SL, Chiodo VA, Hauswirth WW, Boye SE (2014). Dual adeno-associated virus vectors result in efficient in vitro and in vivo expression of an oversized gene, MYO7A. Hum Gene Ther Methods.

[40] Hirsch ML, Li C, Bellon I, Yin C, Chavala S, Pryadkina M (2013). Oversized AAV transductifon is mediated via a DNA-PKcs-independent, Rad51C-dependent repair pathway. Mol Ther.

[41] Penaud-Budloo M, Le Guiner C, Nowrouzi A, Toromanoff A, Cherel Y, Chenuaud P (2008). Adeno-associated virus vector genomes persist as episomal chromatin in primate muscle. J Virol.

[42] Carvalho LS, Turunen HT, Wassmer SJ, Luna-Velez MV, Xiao R, Bennett J (2017). Evaluating efficiencies of dual AAV approaches for retinal targeting. Front Neurosci.

[43] Yan Z, Sun X, Feng Z, Li G, Fisher JT, Stewart ZA (2015). Optimization of recombinant adeno-associated virus-mediated expression for large transgenes, using a synthetic promoter and tandem array enhancers. Hum Gene Ther.

[44] Chew WL, Tabebordbar M, Cheng JK, Mali P, Wu EY, Ng AH (2016). A multifunctional AAV-CRISPR-Cas9 and its host response. Nat Methods.

[45] Chung SH, Mollhoff IN, Nguyen U, Nguyen A, Stucka N, Tieu E (2020). Factors impacting efficacy of AAV-mediated CRISPR-based genome editing for treatment of choroidal neovascularization. Mol Ther Methods Clin Dev.

[46] Stone D, Long KR, Loprieno MA, De Silva Feelixge HS, Kenkel EJ, Liley RM (2021). CRISPR-Cas9 gene editing of hepatitis B virus in chronically infected humanized mice. Mol Ther Methods Clin Dev.

[47] Li H, Sheng C, Liu H, Wang S, Zhao J, Yang L (2018). Inhibition of HBV expression in HBV transgenic mice using AAV-delivered CRISPR-SaCas9. Front Immunol.

[48] Scott T, Moyo B, Nicholson S, Maepa MB, Watashi K, Ely A (2017). ssAAVs containing cassettes encoding SaCas9 and guides targeting hepatitis B virus inactivate replication of the virus in cultured cells. Sci Rep.

[49] Kim N, Kim HK, Lee S, Seo JH, Choi JW, Park J (2020). Prediction of the sequence-specific cleavage activity of Cas9 variants. Nat Biotechnol.

[50] McCarty DM, Monahan PE, Samulski RJ (2001). Self-complementary recombinant adeno-associated virus (scAAV) vectors promote efficient transduction independently of DNA synthesis. Gene Ther.

[51] Zincarelli C, Soltys S, Rengo G, Rabinowitz JE (2008). Analysis of AAV serotypes 1–9 mediated gene expression and tropism in mice after systemic injection. Mol Ther.

[52] Nathwani AC, Gray JT, Ng CY, Zhou J, Spence Y, Waddington SN (2006). Self-complementary adeno-associated virus vectors containing a novel liver-specific human factor IX expression cassette enable highly efficient transduction of murine and nonhuman primate liver. Blood.

[53] Nathwani AC, Reiss UM, Tuddenham EGD, Rosales C, Chowdary P, McIntosh J (2014). Long-term safety and efficacy of factor IX gene therapy in hemophilia B. N Engl J Med.

[54] Chuah MK, Petrus I, De Bleser P, Le Guiner C, Gernoux G, Adjali O (2014). Liver-specific transcriptional modules identified by genome-wide in silico analysis enable efficient gene therapy in mice and non-human primates. Mol Ther J Am Soc Gene Ther.

[55] Hinderer C, Katz N, Buza EL, Dyer C, Goode T, Bell P (2018). Severe toxicity in nonhuman primates and piglets following high-dose intravenous administration of an adeno-associated virus vector expressing human SMN. Hum Gene Ther.

[56] Calcedo R, Morizono H, Wang L, McCarter R, He J, Jones D (2011). Adeno-associated virus antibody profiles in newborns, children, and adolescents. Clin Vaccine Immunol.

[57] Fitzpatrick Z, Leborgne C, Barbon E, Masat E, Ronziti G, van Wittenberghe L (2018). Influence of pre-existing anti-capsid neutralizing and binding antibodies on AAV vector transduction. Mol Ther.

[58] Li C, He Y, Nicolson S, Hirsch M, Weinberg MS, Zhang P (2013). Adeno-associated virus capsid antigen presentation is dependent on endosomal escape. J Clin Investig.

[59] Martino AT, Basner-Tschakarjan E, Markusic DM, Finn JD, Hinderer C, Zhou S (2013). Engineered AAV vector minimizes in vivo targeting of transduced hepatocytes by capsid-specific CD8+ T cells. Blood.

[60] Pien GC, Basner-Tschakarjan E, Hui DJ, Mentlik AN, Finn JD, Hasbrouck NC (2009). Capsid antigen presentation flags human hepatocytes for destruction after transduction by adeno-associated viral vectors. J Clin Investig.

[61] Zhong L, Li B, Jayandharan G, Mah CS, Govindasamy L, Agbandje-McKenna M (2008). Tyrosine-phosphorylation of AAV2 vectors and its consequences on viral intracellular trafficking and transgene expression. Virology.

[62] Tse LV, Klinc KA, Madigan VJ, Castellanos Rivera RM, Wells LF, Havlik LP (2017). Structure-guided evolution of antigenically distinct adeno-associated virus variants for immune evasion. Proc Natl Acad Sci U S A.

[63] Tseng Y-S, Gurda BL, Chipman P, McKenna R, Afione S, Chiorini JA (2015). Adeno-associated virus serotype 1 (AAV1)- and AAV5-antibody complex structures reveal evolutionary commonalities in parvovirus antigenic reactivity. J Virol.

[64] Davidsson M, Wang G, Aldrin-Kirk P, Cardoso T, Nolbrant S, Hartnor M (2019). A systematic capsid evolution approach performed in vivo for the design of AAV vectors with tailored properties and tropism. Proc Natl Acad Sci.

[65] Körbelin J, Sieber T, Michelfelder S, Lunding L, Spies E, Hunger A (2016). Pulmonary targeting of adeno-associated viral vectors by next-generation sequencing-guided screening of random capsid displayed peptide libraries. Mol Ther J Am Soc Gene Ther.

[66] Deverman BE, Pravdo PL, Simpson BP, Kumar SR, Chan KY, Banerjee A (2016). Cre-dependent selection yields AAV variants for widespread gene transfer to the adult brain. Nat Biotechnol.

[67] Hatakeyama S, Matsumoto M, Nakayama KI (2005). Mapping of ubiquitination sites on target proteins. Methods Enzymol.

[68] Vigerust DJ, Shepherd VL (2007). Virus glycosylation: role in virulence and immune interactions. Trends Microbiol.

[69] Maarifi G, Fernandez J, Portilho DM, Boulay A, Dutrieux J, Oddos S (2018). RanBP2 regulates the anti-retroviral activity of TRIM5α by SUMOylation at a predicted phosphorylated SUMOylation motif. Commun Biol.

[70] Zhang T, Ye Z, Yang X, Qin Y, Hu Y, Tong X (2017). NEDDylation of PB2 reduces its stability and blocks the replication of influenza A virus. Sci Rep.

[71] Mary B, Maurya S, Kumar M, Bammidi S, Kumar V, Jayandharan GR (2019). Molecular engineering of adeno-associated virus capsid improves its therapeutic gene transfer in murine models of hemophilia and retinal degeneration. Mol Pharm.

[72] Maurya S, Mary B, Jayandharan GR (2020). Improved ocular gene transfer with a Neddylation-site modified AAV-RPE65 vector in rd12 mice. Eye.

[73] D'Souza AA, Devarajan PV (2015). Asialoglycoprotein receptor mediated hepatocyte targeting—strategies and applications. J Control Release.

[74] Mével M, Bouzelha M, Leray A, Pacouret S, Guilbaud M, Penaud-Budloo M (2020). Chemical modification of the adeno-associated virus capsid to improve gene delivery. Chem Sci.

[75] Lisowski L, Dane AP, Chu K, Zhang Y, Cunningham SC, Wilson EM (2014). Selection and evaluation of clinically relevant AAV variants in a xenograft liver model. Nature.

[76] Pei X, Shao W, Xing A, Askew C, Chen X, Cui C (2020). Development of AAV variants with human hepatocyte tropism and neutralizing antibody escape capacity. Mol Ther Methods Clin Dev.

[77] Paulk NK, Pekrun K, Zhu E, Nygaard S, Li B, Xu J (2018). Bioengineered AAV capsids with combined high human liver transduction in vivo and unique humoral seroreactivity. Mol Ther.

[78] Asuri P, Bartel MA, Vazin T, Jang JH, Wong TB, Schaffer DV (2012). Directed evolution of adeno-associated virus for enhanced gene delivery and gene targeting in human pluripotent stem cells. Mol Ther.

[79] Qian R, Xiao B, Li J, Xiao X (2021). Directed evolution of AAV serotype 5 for increased hepatocyte transduction and retained low humoral seroreactivity. Mol Ther Methods Clin Dev.

[80] Santiago-Ortiz J, Ojala DS, Westesson O, Weinstein JR, Wong SY, Steinsapir A (2015). AAV ancestral reconstruction library enables selection of broadly infectious viral variants. Gene Ther.

[81] Carvalho LS, Xiao R, Wassmer SJ, Langsdorf A, Zinn E, Pacouret S (2018). Synthetic adeno-associated viral vector efficiently targets mouse and nonhuman primate retina in vivo. Hum Gene Ther.

[82] Gu X, Chai R, Luo G, Dong B, Li W, Yilai S (2019). Transduction of adeno-associated virus vectors targeting hair cells and supporting cells in the neonatal mouse cochlea. Front Cell Neurosci.

[83] Hu C-J, Lu Y-C, Tsai Y-H, Cheng H-Y, Takeda H, Huang C-Y (2020). Efficient in utero gene transfer to the mammalian inner ears by the synthetic adeno-associated viral vector Anc80L65. Mol Ther Methods Clin Dev.

[84] Hudry E, Andres-Mateos E, Lerner EP, Volak A, Cohen O, Hyman BT (2018). Efficient gene transfer to the central nervous system by single-stranded Anc80L65. Mol Ther Methods Clin Dev.

[85] Landegger LD (2017). A synthetic AAV vector enables safe and efficient gene transfer to the mammalian inner ear. Nat Biotechnol.

[86] Zinn E, Pacouret S, Khaychuk V, Turunen HT, Carvalho LS, Andres-Mateos E (2015). In silico reconstruction of the viral evolutionary lineage yields a potent gene therapy vector. Cell Rep.

[87] Gao GP, Lu Y, Sun X, Johnston J, Calcedo R, Grant R (2006). High-level transgene expression in nonhuman primate liver with novel adeno-associated virus serotypes containing self-complementary genomes. J Virol.

[88] Nathwani AC, Gray JT, McIntosh J, Ng CY, Zhou J, Spence Y (2007). Safe and efficient transduction of the liver after peripheral vein infusion of self-complementary AAV vector results in stable therapeutic expression of human FIX in nonhuman primates. Blood.

[89] Chan K, Sterling JF, Roberts SA, Bhagwat AS, Resnick MA, Gordenin DA (2012). Base damage within single-strand DNA underlies in vivo hypermutability induced by a ubiquitous environmental agent. PLoS Genet.

[90] Chen CC, Ko TM, Ma HI, Wu HL, Xiao X, Li J (2007). Long-term inhibition of hepatitis B virus in transgenic mice by double-stranded adeno-associated virus 8-delivered short hairpin RNA. Gene Ther.

[91] Grimm D, Streetz KL, Jopling CL, Storm TA, Pandey K, Davis CR (2006). Fatality in mice due to oversaturation of cellular microRNA/short hairpin RNA pathways. Nature.

[92] Ye L, Kan F, Yan T, Cao J, Zhang L, Wu Z (2017). Enhanced antiviral and antifibrotic effects of short hairpin RNAs targeting HBV and TGF-beta in HBV-persistent mice. Sci Rep.

[93] Mockenhaupt S, Grosse S, Rupp D, Bartenschlager R, Grimm D (2015). Alleviation of off-target effects from vector-encoded shRNAs via codelivered RNA decoys. Proc Natl Acad Sci U S A.

[94] Grimm D, Wang L, Lee JS, Schurmann N, Gu S, Borner K (2010). Argonaute proteins are key determinants of RNAi efficacy, toxicity, and persistence in the adult mouse liver. J Clin Investig.

[95] Yan K, Feng J, Liu X, Wang H, Li Q, Li J (2021). Inhibition of hepatitis B virus by AAV8-derived CRISPR/SaCas9 expressed from liver-specific promoters. Front Microbiol.

[96] Weber ND, Stone D, Sedlak RH, Feelixge HSDS, Roychoudhury P, Schiffer JT (2014). AAV-mediated delivery of zinc finger nucleases targeting hepatitis B virus inhibits active replication. PLoS ONE.

[97] Pillay S, Zou W, Cheng F, Puschnik AS, Meyer NL, Ganaie SS (2017). Adeno-associated virus (AAV) serotypes have distinctive interactions with domains of the cellular AAV receptor. J Virol.

[98] Pillay S, Meyer NL, Puschnik AS, Davulcu O, Diep J, Ishikawa Y (2016). An essential receptor for adeno-associated virus infection. Nature.

[99] Wieland SF (2015). The chimpanzee model for hepatitis B virus infection. Cold Spring Harb Perspect Med.

[100] Fu L, Hu H, Liu Y, Jing Z, Li W (2017). Woodchuck sodium taurocholate cotransporting polypeptide supports low-level hepatitis B and D virus entry. Virology.

[101] Guo WN, Zhu B, Ai L, Yang DL, Wang BJ (2018). Animal models for the study of hepatitis B virus infection. Zool Res.

[102] Xia Y, Carpentier A, Cheng X, Block PD, Zhao Y, Zhang Z (2017). Human stem cell-derived hepatocytes as a model for hepatitis B virus infection, spreading and virus–host interactions. J Hepatol.

[103] Winer BY, Huang T, Low BE, Avery C, Pais MA, Hrebikova G (2017). Recapitulation of treatment response patterns in a novel humanized mouse model for chronic hepatitis B virus infection. Virology.

[104] Sun S, Li J (2017). Humanized chimeric mouse models of hepatitis B virus infection. Int J Infect Dis.

[105] Dandri M, Burda MR, Torok E, Pollok JM, Iwanska A, Sommer G (2001). Repopulation of mouse liver with human hepatocytes and in vivo infection with hepatitis B virus. Hepatology.

[106] Chisari FV (1995). Hepatitis B virus transgenic mice: insights into the virus and the disease. Hepatology.

[107] Chisari FV (1996). Hepatitis B virus transgenic mice: models of viral immunobiology and pathogenesis. Curr Top Microbiol Immunol.

[108] Marion P, Salazar F, Liittschwager K, Bordier B, Seeger C, Winters M, Schinazi RF, Sommadossi J-P, Rice CM (2003). A transgenic mouse lineage useful for testing antivirals targeting hepatitis B virus. Frontiers in viral hepatitis.

[109] Carmona S, Ely A, Crowther C, Moolla N, Salazar FH, Marion PL (2006). Effective inhibition of HBV replication in vivo by anti-HBx short hairpin RNAs. Mol Ther.

[110] von Freyend MJ, Untergasser A, Arzberger S, Oberwinkler H, Drebber U, Schirmacher P (2011). Sequential control of hepatitis B virus in a mouse model of acute, self-resolving hepatitis B. J Viral Hepat.

[111] Sprinzl MF, Oberwinkler H, Schaller H, Protzer U (2001). Transfer of hepatitis B virus genome by adenovirus vectors into cultured cells and mice: crossing the species barrier. J Virol.

[112] Huang LR, Gabel YA, Graf S, Arzberger S, Kurts C, Heikenwalder M (2012). Transfer of HBV genomes using low doses of adenovirus vectors leads to persistent infection in immune competent mice. Gastroenterology.

[113] Huang YH, Fang CC, Tsuneyama K, Chou HY, Pan WY, Shih YM (2011). A murine model of hepatitis B-associated hepatocellular carcinoma generated by adeno-associated virus-mediated gene delivery. Int J Oncol.

[114] Ye L, Yu H, Li C, Hirsch ML, Zhang L, Samulski RJ (2015). Adeno-associated virus vector mediated delivery of the HBV genome induces chronic hepatitis B virus infection and liver fibrosis in mice. PLoS ONE.

[115] Yang D, Liu L, Zhu D, Peng H, Su L, Fu YX (2014). A mouse model for HBV immunotolerance and immunotherapy. Cell Mol Immunol.

[116] Saeidi A, Zandi K, Cheok YY, Saeidi H, Wong WF, Lee CYQ (2018). T-cell exhaustion in chronic infections: reversing the state of exhaustion and reinvigorating optimal protective immune responses. Front Immunol.

[117] Wherry EJ, Kurachi M (2015). Molecular and cellular insights into T cell exhaustion. Nat Rev Immunol.

[118] Lucifora J, Salvetti A, Marniquet X, Mailly L, Testoni B, Fusil F (2017). Detection of the hepatitis B virus (HBV) covalently-closed-circular DNA (cccDNA) in mice transduced with a recombinant AAV-HBV vector. Antiviral Res.

[119] Ko C, Su J, Festag J, Bester R, Kosinska AD, Protzer U (2021). Intramolecular recombination enables the formation of hepatitis B virus (HBV) cccDNA in mice after HBV genome transfer using recombinant AAV vectors. Antiviral Res.

[120] Burwitz BJ, Wettengel JM, Muck-Hausl MA, Ringelhan M, Ko C, Festag MM (2017). Hepatocytic expression of human sodium-taurocholate cotransporting polypeptide enables hepatitis B virus infection of macaques. Nat Commun.

[121] Biswas S, Stanton J, Hashiguchi P, Bimber BN, Protzer U, Sacha JB, et al., editors. A rhesus macaque model of chronic hbv infection for cure research. In: Conference on retroviruses and opportunistic infections. Boston. 2020.

[122] Carmona S, Jorgensen MR, Kolli S, Crowther C, Salazar FH, Marion PL (2009). Controlling HBV replication in vivo by intravenous administration of triggered PEGylated siRNA-nanoparticles. Mol Pharm.

[123] Burwitz BJ, Wettengel JM, Muck-Hausl MA, Ringelhan M, Ko C, Festag MM (2017). Hepatocytic expression of human sodium-taurocholate cotransporting polypeptide enables hepatitis B virus infection of macaques. Nat Commun.

